# LAG-3 expression in the inflammatory microenvironment of glioma

**DOI:** 10.1007/s11060-021-03721-x

**Published:** 2021-03-02

**Authors:** Maximilian J. Mair, Barbara Kiesel, Katharina Feldmann, Georg Widhalm, Karin Dieckmann, Adelheid Wöhrer, Leonhard Müllauer, Matthias Preusser, Anna S. Berghoff

**Affiliations:** 1 Division of Oncology and Christian Doppler Laboratory for Personalized Immunotherapy, Department of Medicine I, Medical University of Vienna, Waehringer Guertel 18-20, 1090 Vienna, Austria; 2 Department of Neurosurgery, Medical University of Vienna, Vienna, Austria; 3 Department of Radiation Oncology, Medical University of Vienna, Vienna, Austria; 4 Division of Neuropathology and Neurochemistry, Department of Neurology, Medical University of Vienna, Vienna, Austria; 5 Department of Pathology, Medical University of Vienna, Vienna, Austria

**Keywords:** Glioma, Glioblastoma, Tumor microenvironment, LAG-3, Immune checkpoint

## Abstract

**Purpose:**

Immune modulatory therapies including immune checkpoint inhibitors have so far failed to result in clinically meaningful efficacy in glioma. We aimed to investigate lymphocyte activation gene 3 (LAG-3), an inhibitory receptor on immune cells and target of second-generation immune checkpoint inhibitors, in glioma.

**Methods:**

97 patients with diffuse glioma (68 with glioblastoma, 29 with WHO grade II-III glioma) were identified from the Neuro-Biobank of the Medical University of Vienna. LAG-3 expression in the inflammatory microenvironment was assessed by immunohistochemistry (monoclonal anti-LAG-3 antibody, clone 17B4) and correlated to CD3+ , CD8+ , CD20+ and PD-1+ tumor-infiltrating lymphocytes (TILs) and PD-L1 expression on tumor cells.

**Results:**

LAG-3+ TILs could be observed in 10/97 (10.3%) IDH-wildtype samples and in none of the included IDH-mutant glioma samples (p = 0.057). Further, LAG-3+ TILs were only observed in WHO grade IV glioblastoma, while none of the investigated WHO grade II–III glioma presented with LAG-3+ TILs (p = 0.03). No association of O6-methylguanine-DNA-methyltransferase (MGMT) promoter methylation and presence of LAG-3+ TILs was observed (p = 0.726). LAG-3 expression was associated with the presence of CD3+ (p = 0.029), CD8+ (p = 0.001), PD-1+ (p < 0.001) TILs and PD-L1+ tumor cells (p = 0.021), respectively. No association of overall survival with LAG-3+ TIL infiltration was evident (median OS 9.9 vs. 14.2 months, p = 0.95).

**Conclusions:**

LAG-3 is only rarely expressed on TILs in IDH-wildtype glioma and associated with active inflammatory milieu as defined by higher TIL density. Immune microenvironment diversity should be considered in the design of future immunotherapy trials in glioma.

**Supplementary Information:**

The online version contains supplementary material available at 10.1007/s11060-021-03721-x.

## Introduction

Immune checkpoint inhibitors are a major breakthrough in oncology as durable responses can be observed in a variety of solid malignancies. However, despite the efficacy in secondary brain tumors [1–3], no clinical benefit was so far observed in primary brain tumors such as glioma. The CheckMate-143 trial comparing the anti-PD-L1 agent nivolumab to bevacizumab in recurrent glioblastoma did not meet its primary endpoint, as overall survival was comparable in both treatment arms [4]. Similarly, two trials investigating the activity of PD-1 inhibitors in newly diagnosed glioblastoma failed to meet their primary endpoints according to recently published press releases, although the final publications are pending [5, 6]. Interestingly, membranous PD-L1 expression was observed in 37.6–61% of human glioblastoma samples, while diffuse/fibrillary PD-L1 expression was seen in 88.0% of patients, indicating that the target is present in the majority of patients [7, 8]. Still, the objective response rate of 7.8% towards nivolumab treatment was limited in recurrent glioblastoma [4], underlining the need to explore new immune modulatory treatment targets.

Lymphocyte activation gene 3 (LAG-3) is an inhibitory receptor which is mainly found on activated immune cells [9]. Frequently co-expressed with PD-1 on exhausted T cells, LAG-3 has become an interesting target for immune-modulating agents either alone or in combination with inhibitors of the PD-1/PD-L1 axis [10]. Anti-LAG-3 agents are under investigation in phase I–III trials in a wide array of solid tumors including lung, gastric, head and neck, hepatocellular and renal cancer as well as lymphoma and melanoma [11]. In the latter, LAG-3 expression was associated with a higher objective response rate towards LAG-3 blockade in early data of a phase I/IIA trial [12]; however, the predictive value of LAG-3 expression towards response to LAG-3 immune checkpoint inhibitors is still not fully elucidated.

In glioma, a preclinical study using a syngenic mouse model showed that treatment with anti-LAG-3 antibodies either alone or in combination with PD-1 inhibition is effective and results in prolonged survival [13]. However, systematic data on LAG-3 expression in human glioma tissue are missing so far. Therefore, the aim of this study was to evaluate LAG-3 expression in the tumor microenvironment of adult glioblastoma and WHO grade II–III glioma cases.

## Materials and methods

### Patient cohort

Patients aged ≥ 18 years at diagnosis who were treated for WHO grade II–III glioma or glioblastoma were identified from the Neuro-Biobank of the Medical University of Vienna. Histological diagnosis was performed by a board-certified neuropathologist according to the 2016 WHO Classification of Central Nervous Tumours [14]. Patient data were stored in a password-secured database (FileMaker Pro Advanced/Server 17, FileMaker Inc., Santa Clara, USA) and were handled anonymously. This study was performed according to the ethical standards of the Ethics Committee of the Medical University of Vienna (approval no. 1166/2019) and the Helsinki Declaration of 1964 with all its amendments.

### Immunohistochemistry

Immunohistochemical analysis was executed using a Ventana Benchmark Ultra immunostainer (Roche Ventana Medical Systems, Tucson, AZ, USA) as described previously [15]. Used antibodies are listed in Supplementary Table 1. Analysis of CD3+ , CD8+ , CD20+ and PD-1 + TILs as well as PD-L1 expression on tumor cells was evaluated semiquantitatively and available from previous publications [8, 16]. LAG-3+ TIL were also evaluated semiquantitatively by overall impression at low microscopic magnification (×100). Further, accumulation of TILs in predefined areas (within the viable tumor tissue, in the perivascular region and, if applicable, in the invasion zone to the surrounding brain parenchyma) was analyzed as higher magnification (×200–×400). Previously published criteria were used to describe TIL density as sparse, moderate, dense or very dense [8, 16].

### Statistical analysis

Independence of categorical variables was assessed using Chi-square or Fisher’s exact test as appropriate. Overall survival (OS) was defined as the time between first surgery and all-cause death and was compared applying the log-rank test. Results were considered significant at a p value of ≤ 0.05. Due to the hypothesis-generating study design, no correction for multiple testing was applied [17].

Statistical analysis was performed using GraphPad Prism 8 (La Jolla, CA, USA), R 3.6.1 (The R Foundation for Statistical Computing, Vienna, Austria) with RStudio 1.2.1335 (RStudio Inc., Boston, MA, USA) and the packages “ggplot2” (version 3.2.0), “GGally” (version 1.4.0), “VennDiagram” (version 1.6.20), “survival” (version 2.44–1.1) and “ggpubr” (version 0.2.3).

## Results

### Patients’ characteristics

97 patients were included, including 68/97 (70.1%) patients with glioblastoma and 29/97 (29.9%) with WHO grade II–III glioma. Isocitrate dehydrogenase (IDH) mutations could be detected in 27/97 (27.8%) of included samples, while 70/97 (72.2%) specimens were IDH wildtype (IDH-wt). Further baseline characteristics of the studied cohort are given in Table 1.

**Table 1:** Patients’ characteristics

	n = 97
Gender	
Male	56 (57.7%)
Female	41 (42.3%)
Median age at diagnosis (range)	55 (25–80)
WHO grade	
WHO II	21 (21.7%)
WHO III	8 (8.2%)
WHO IV	68 (70.1%)
IDH mutation	
IDH mutated	27 (27.8%)
IDH wildtype	70 (72.2%)
MGMT promoter methylation	
Methylated	23 (23.7%)
Unmethylated	36 (37.1%)
Unknown	38 (39.2%)
Diagnosis according to WHO 2016 classification	
Diffuse astrocytoma, IDH-mt	11 (11.3%)
Gemistocytic astrocytoma, IDH-mt	1 (1.0%)
Anaplastic astrocytoma, IDH-mt	2 (2.1%)
Diffuse astrocytoma, IDH-wt	2 (2.1%)
Oligodendroglioma, IDH-mt, 1p19q-codeleted	7 (7.2%)
Anaplastic oligodendroglioma, IDH-mt, 1p19q-codeleted	6 (6.2%)
Glioblastoma, IDH-wt	68 (69.4%)

### LAG-3 expression on tumor-infiltrating lymphocytes

**Fig. 1:**
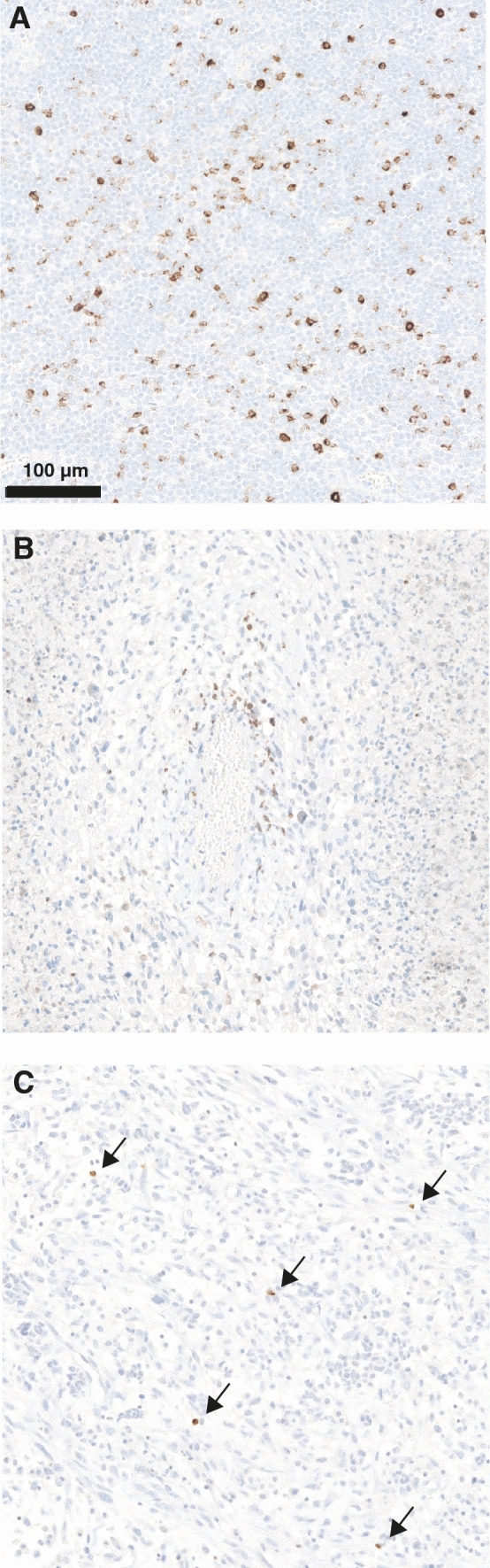
LAG-3 staining in **a** tonsil as positive control, **b** perivascular region and **c** tumor tissue in glioma. Magnification ×200, scale bar 100 µm

LAG-3 expression on TILs in glioma was observed in 10/97 (10.3%) cases (Fig. 1; tonsil as positive control shown in Fig. 1a). As previously described with other TIL subsets [16], also LAG-3+ TILs accumulated in the perivascular region, whereas the density of LAG-3+ TILs was sparse in tumor tissue (Fig. 1b/c).

**Fig. 2:**
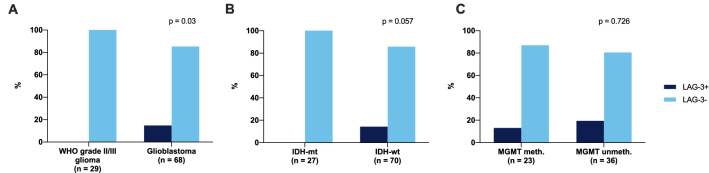
Fractions of LAG-3+ cases according to **a** WHO grade, **b** IDH mutational status and **c** MGMT promoter methylation status. P-values as determined by Fisher’s exact test

LAG-3+ TILs were observed in 10/68 (14.7%) of glioblastoma samples while no tissue specimen of WHO grade II-III gliomas showed LAG-3+ TIL infiltration (p = 0.03, Fisher’s exact test). Similarly, all LAG-3+ samples were from IDH-wildtype tumors (10/70, 14.3%) while no LAG-3+ TILs could be found in IDH-mutant glioma (p = 0.057, Fisher’s exact test). In terms of O6-methylguanine-DNA-methyltransferase (MGMT) promoter methylation, 3/23 (13.0%) MGMT promoter-methylated tumors showed LAG-3+ TIL infiltration as compared to 7/36 (19.4%) unmethylated specimen (p = 0.726, Fisher’s exact test; Fig. 2).

There were no associations of LAG-3+ TIL infiltration with age at first surgery (p = 0.135, Mann–Whitney-U test) and gender (p = 0.183, Fisher’s exact test).

### Correlation of LAG-3+ TILs with other TIL subsets

CD3+ TILs were observed in 65/97 (67.0%) samples, while CD8 + TILs and PD-1 + TILs were seen in 39/97 (40.2%) and 14/97 (14.4%) specimen, respectively. Furthermore, PD-L1 expression on tumor cells was detected in 43/97 (44.3%) tumors.

**Fig. 3:**
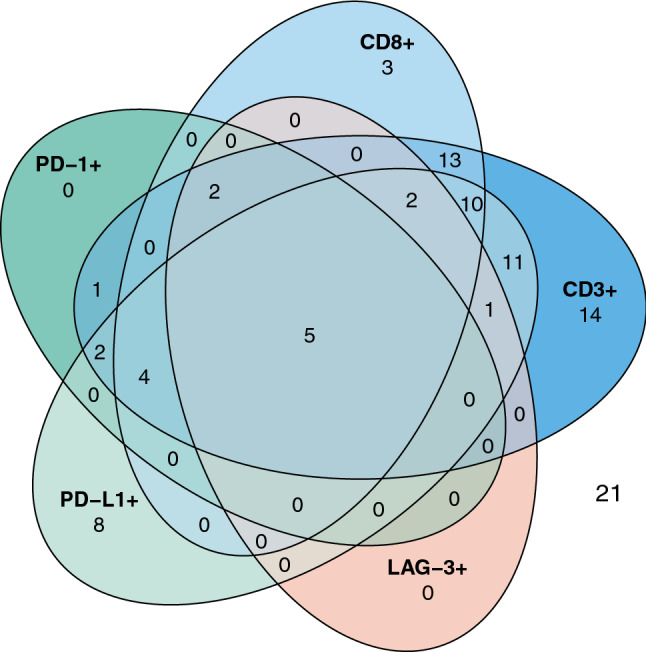
Venn diagram showing the concordance of tissue-based immune markers in the included samples of the overall cohort

Only samples with CD3+ TIL infiltration were also positive for LAG-3+ TILs (10/65 CD3+ vs. 0/32 CD3-; p = 0.029, Fisher’s exact test; Table 2A). In 9/30 (30.0%) CD8+ samples also LAG-3+ TIL infiltration was observed, while only 1/58 (1.7%) of CD8- specimen showed LAG-3+ TIL infiltration (p = 0.001, Fisher’s exact test). 7/14 (50.0%) PD-1+ specimen showed LAG-3+ TIL infiltration while only 3/83 (3.6%) PD-1− samples presented with LAG-3+ TILs (p < 0.001, Fisher’s exact test). An association between PD-L1 expression on tumor cells and LAG-3+ TIL infiltration was observed, as 8/43 (18.6%) samples with membranous PD-L1 expression on tumor cells were infiltrated by LAG-3+ lymphocytes in contrast to only 2/54 (3.7%) in PD-L1− cases (p = 0.021, Fisher’s exact test). A Venn diagram in Fig. 3 illustrates the co-occurrence of CD3+ , CD8+ , PD-1+ and LAG-3+ TILs as well as PD-L1+ tumor cells in the overall cohort. Of note, 21/97 (21.6%) cases did show neither of the analyzed immune cell subsets.

**Table 2:** Association between LAG-3 expression and other tumor-infiltrating lymphocyte subsets in (A) the overall cohort and (B) the IDH-wt glioblastoma cohort

(A)	Overall cohort	LAG-3		
	n = 97	+	−	
CD3	+	10	55	**p = 0.029**
	−	0	32	
CD8	+	9	30	**p = 0.001**
	−	1	57	
PD-1	+	7	7	**p < 0.001**
	−	3	80	
PD-L1	+	8	35	**p = 0.021**
	−	2	52	
	Median (range)	1% (0–70%)	0% (0–60%)	p = 0.052

(B)	IDH-wt glioblastoma	LAG-3		
	n = 68	+	−	
CD3	+	10	38	**p = 0.020**
	−	0	20	
CD8	+	9	19	**p < 0.001**
	−	1	39	
CD20	+	6	12	**p = 0.017**
	−	4	46	
PD-1	+	7	7	**p < 0.001**
	−	3	51	
PD-L1	+	8	32	p = 0.179
	−	2	26	
	Median (range)	1% (0–70%)	1% (0–60%)	p = 0.436

P-values as determined by Fisher’s exact test

Bold p-values indicate p ≤ 0.05

### Correlation of LAG-3+ TILs with CD3+ , CD8+ , CD20+ , PD-1+ TIL infiltration and PD-L1 expression on tumor cells in the IDH-wt glioblastoma cohort

In IDH-wt glioblastoma, CD3+ TILs were observed in 48/68 (70.6%) samples, while CD8+ TILs were seen in 28/68 (41.2%), CD20+ in 18/68 (26.5%) and PD-1+ TILs in 14/68 (20.6%) samples. PD-L1 expression on tumor cells was seen in 40/68 (58.8%) cases.

Again, only samples with CD3+ TIL infiltration had LAG-3+ TILs (10/48 CD3+ vs. 0/20 CD3-; p = 0.028, Fisher’s exact test, Table 2B). In 9/28 (32.1%) CD8+ cases LAG-3+ TIL infiltration was seen, while only 1/40 (2.5%) of CD8- samples had LAG-3+ TILs (p < 0.001, Fisher’s exact test). Similarly, in 6/18 (33.3%) CD20+ tumors an infiltration with LAG-3+ TILs was observed, while this was only the case in 4/46 (8.7%) CD20- samples (p = 0.017, Fisher’s exact test). Moreover, 7/14 (50.0%) of tumors with PD-1+ immune cell infiltration showed LAG-3+ TILs, while in only 3/54 (5.6%) PD-1− tumors an infiltration with LAG-3+ lymphocytes was seen (p < 0.001, Fisher’s exact test). However, no correlation between membranous PD-L1 expression on tumor cells and LAG-3+ TILs could be detected (8/40 (20.0%) PD-1+ vs. 2/28 (7.1%); p = 0.179, Fisher’s exact test).

### Prognostic impact of LAG-3+ TILs in glioblastoma

**Fig. 4:**
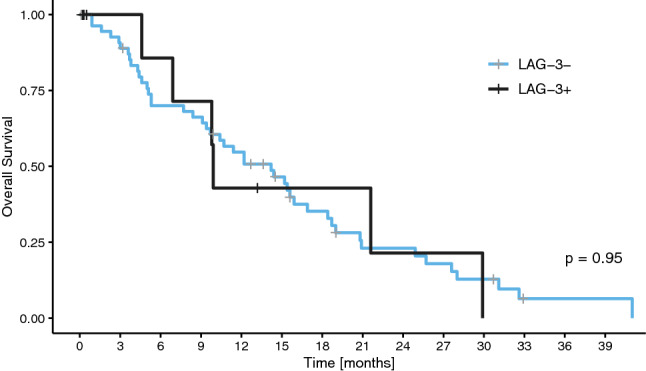
Overall survival in the glioblastoma cohort according to LAG-3 expression in the tumor microenvironment

As only IDH-wt glioblastoma cases had LAG-3+ TILs, survival analysis according to LAG-3 expression was only performed in this subgroup to exclude the prognostic impact of different glioma subgroups. However, there was no significant difference in overall survival between patients with and without LAG-3+ TIL infiltration (median OS 9.9 months vs. 14.2 months; p = 0.95, log-rank test; Fig. 4).

## Discussion

Here, we investigated LAG-3 expression in the inflammatory microenvironment of glioma. We found that only a small subset of tissue samples exhibited sparse infiltration by LAG-3+ TILs, and all of them were IDH-wt glioblastoma cases. Overlap of LAG-3+ TILs infiltration with other immune markers was observed, underscoring that LAG-3 is expressed in samples with a particularly active immune microenvironment. The present study therefore underlines the diversity of the immune microenvironment composition in glioma. Future studies on immune modulating therapies should address the immune microenvironment diversity of glioma.

In our cohort, only ~ 10% of samples showed sparse LAG-3+ TIL infiltration. In line with our results, a transcriptomic study based on The Cancer Genome Atlas (TCGA) data showed that glioblastomas (and even more distinctly low-grade gliomas) were among the tumors with the lowest expression of LAG-3 as compared to other malignancies [18]. In contrast, Harris-Bookman et al. observed that 66% of glioblastoma samples in a small cohort of 9 cases displayed variable LAG-3 expression on perivascular lymphocytes while no WHO grade II-III glioma or IDH-mt cases were analyzed [13].

LAG-3+ TIL infiltration was associated with CD3+ , CD8+ and PD-1+ TILs as well as PD-L1+ tumor cells and therefore more frequently observed in specimens with an active inflammatory microenvironment. Indeed, previous studies in ovarian cancer also suggested a correlation of LAG-3+ TIL density with PD-1+ TIL density and therefore a marker for increased cancer-immune system interaction [19]. Our data therefore underline the heterogenous composition of the glioma inflammatory microenvironment cohort also in terms of LAG-3+ TIL density. The so far conducted clinical trials on LAG-3 immune checkpoint inhibitor inhibition did not require a particular biomarker. However, previous studies from extracranial tumors have repetitively underscored that an active inflammatory microenvironment is needed for a clinically meaningful tumor response [20]. Indeed, the combination of anti-PD-1 with anti-LAG-3 antibodies has not been not shown to be superior than either treatment alone in a syngeneic glioma mouse model [13]. However, no further analysis on the inflammatory microenvironment or the presence of LAG-3+ TILs was included. A phase I trial is currently investigating the combination of nivolumab with the anti-LAG-3 agent relatlimab (NCT02658981) in recurrent glioblastoma. In the interim analysis, the median OS in the LAG-3 only and combination arms was 8.5 and 8 months, respectively [21]. However, with 7 still living patients in the combination arm and 3 out of 7 with a survival beyond 20 months, durable responses might be seen in a small subset of patients. Nevertheless, also in this preliminary trial no inflammatory biomarkers were mandatory for inclusion and resistance of immunologically cold tumors could bias the result. Indeed, in extracranial tumors, LAG-3 expression in melanoma was shown to be associated with better prognosis and response to immune checkpoint inhibitors [22]; however, further translational data from trials investigating LAG-3-blocking agents in solid tumors are needed.

Our study has several limitations. First, the retrospective design is inherently linked to heterogenous baseline characteristics in the studied cohort. Furthermore, only few patients with WHO grade II-III glioma and IDH-mutated glioma could be included which impeded further testing for statistical effects in these subgroups. Due to the low rate of LAG-3+ samples, further correlative analyses could not be performed due to limited statistical power. Still, data on LAG-3 expression in glioma patients is rare and the here presented cohort is among the largest published so far.

In conclusion, LAG-3 is only expressed in a small number of human glioma samples. The expression of LAG-3+ in the inflammatory microenvironment shows considerable heterogeneity that needs to be acknowledged in clinical trial planning. Further profiling of tumor – immune system interactions is warranted in glioma, as only a small subset of patients responded in so far conducted immunotherapy trials and reliable biomarkers to identify benefitting patients from immune modulatory treatment modalities including LAG-3 blockade are still needed.

## Supplementary Information

Below is the link to the electronic supplementary material.

Electronic supplementary material 1 (DOCX 13 kb)
