# Supplementary material for: LAG-3 expression in the inflammatory microenvironment of glioma
Source: J Neurooncol. 2021 Mar 2;152(3):533–9. doi: 10.1007/s11060-021-03721-x (PMC8084780; doi:10.1007/s11060-021-03721-x)
Supplement: Supplementary file 1 — Electronic supplementary material 1 (DOCX 13 kb) [file 11060_2021_3721_MOESM1_ESM.docx]

**Supplementary Tables**

**Supplementary Table 1. Antibodies used for immunohistochemistry analysis.**

| **Marker** | **Antibody Clone** | **Company** | **Dilution** |
| --- | --- | --- | --- |
| CD3 | Monoclonal rabbit antibody, Clone SP7 / Ref. Nr. RM9107-S1 | Thermo Fisher Scientific, Cheshire, UK | 1:200 |
| CD8 | Monoclonal mouse antibody, anti-Human CD8/ Clone C8/144B/ M7103 | DakoCytomation  Glostrup, Denmark | 1:100 |
| CD20 | Monoclonal mouse antibody, Clone CD20cy/Clone L26/M0755 | DakoCytomation  Glostrup, Denmark | 1:400 |
| PD-1 | Monoclonal mouse antibody, ab52587 | Abcam, Cambridge, UK | 1:100 |
| PD-L1 | Monoclonal mouse antibody, clone 5H1 (kindly provided by Dr. Lieping Chen) | Yale University, New Naven, CT, USA | 1:100 |
| LAG-3 | Monoclonal mouse antibody, Clone 17B4 | LSBio, Seattle, WA, USA | 1:100 |
